# Synergistic Effects of Hydrocolloid Combinations on Gluten‐Free Batter and Bread Characteristics

**DOI:** 10.1002/fsn3.71107

**Published:** 2025-10-21

**Authors:** Mahan Parsamajd, Mahboubeh Fazaeli, Marjan Majdinasab, Mohammad‐Taghi Golmakani

**Affiliations:** ^1^ Department of Food Science and Technology School of Agriculture, Shiraz University Shiraz Iran

**Keywords:** amino acid profile, gluten‐free, hydrocolloids, rheological properties, texture analysis, thermal characteristics

## Abstract

Celiac disease and other gluten‐related sensitivities are being identified more often because of better diagnostics and changing diets. The inability to consume gluten‐containing products significantly affects quality of life. This study aimed to improve the characteristics of gluten‐free bread by incorporating hydrocolloids—xanthan gum, guar gum, and hydroxypropyl methylcellulose (HPMC)—individually and in binary combinations at a 2% flour basis. The physicochemical, textural, rheological, thermal, and amino acid profile properties of the resulting gluten‐free batter and bread were analyzed. The results demonstrated that the gluten‐free batter exhibited shear‐thinning viscoelastic behavior, obeying the power‐law equation (*r* ^2^ > 0.97). Thermal analysis revealed elevated gelatinization temperatures (peak temperature: 108.61°C–115.79°C), indicating hydrocolloid‐induced structural modifications. The hydrocolloid blend composition had a significant influence on the textural properties. The HPMC‐Xanthan combination yielded the most favorable results, exhibiting the lowest hardness (1.31 N), highest cohesiveness (0.78), and greatest resilience (0.45). This contrasted with the HPMC‐Guar blend, which produced the hardest and least resilient bread. Amino acid analysis showed high levels of lysine (112.00 mg/kg), though the overall profile did not meet all nutritional requirements, indicating the need for future targeted fortification. Physicochemical assessments showed moisture content ranging from 52.72% to 55.38%, while color analysis revealed significant differences only in lightness (L*) values. These findings showed that hydrocolloids, particularly HPMC‐Xanthan, can improve the structure and function of gluten‐free bread. This study contributes to the development of gluten‐free formulations with improved texture and stability. These improvements address challenges such as staling and poor texture, which are crucial for wider industrial adoption and consumer satisfaction.

## Graphical abstract

This study explores the synergistic effects of hydrocolloid combinations on the rheological, thermal, and baking characteristics of gluten‐free batter and bread. Results show that adding an HPMC‐Xanthan blend significantly enhances batter viscoelastic properties and improves the gelatinization temperature. This optimized batter ultimately produces superior bread with the lowest hardness, highest cohesiveness, and greatest resilience, leading to improved overall quality and extended shelf life.

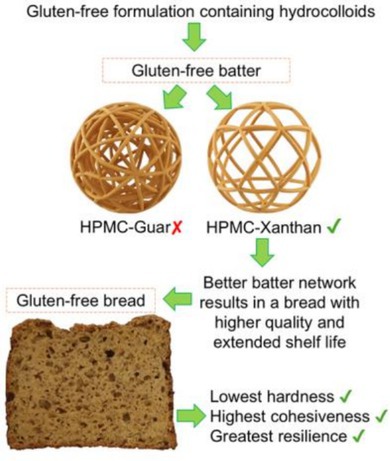

## 1 Introduction

Celiac disease is an autoimmune disorder where patients must strictly avoid all gluten‐containing foods. Its prevalence is rapidly increasing due to lifestyle changes, particularly the rise in consumption of processed, unhealthy foods (Cazac et al. 2024). Improved diagnosis methods and easier patient tracking have also contributed to the growing number of identified cases. Currently, the most effective treatment for celiac disease is a strict, lifelong gluten‐free diet. Grains such as wheat, barley, and rye must be excluded, as their consumption triggers severe symptoms like weight loss, anxiety, nutrient deficiencies, diarrhea, abdominal pain, and fatigue (See and Murray 2006). These symptoms significantly impact the daily life of individuals with celiac disease, and relief can only be achieved through strict adherence to a gluten‐free diet.

However, eliminating gluten from the diet presents challenges, particularly in the quality of gluten‐free products (Demirkesen and Ozkaya 2022). These products are typically high in starch, prone to rapid staling, and often fail to meet the nutritional needs of patients suffering from deficiencies (Woomer and Adedeji 2021). Therefore, improving the core techno‐functional properties, such as texture and shelf‐life, is the critical first step to creating an acceptable product that can later serve as a viable base for nutritional fortification.

Gluten plays a crucial role in dough properties, including extensibility, elasticity, water absorption, and gas‐holding capacity. Without gluten, products often exhibit pale color, hard texture, dry mouthfeel, soft crust, sticky batter, lack of elasticity, and other undesirable traits (Awulachew 2021). To overcome these issues, gluten substitutes must be identified. While several approaches, including the use of dietary fibers, proteins, and starches, have been explored, the most effective strategy so far is the application of hydrocolloids (Irondi et al. 2023; Salehi 2019).

Hydrocolloids are hydrophilic substances composed of long molecular chains, known for their ability to form gels when hydrated (Gao et al. 2024). They are particularly valuable in food systems due to their ability to increase viscosity even at low concentrations. In gluten‐free products, hydrocolloids help improve both rheological and textural properties, making them essential additives in the food industry. Their high molecular weight enables the retention of gas cells during fermentation and baking, mimicking gluten's role in the dough matrix (Gasparre and Rosell 2023). Hydrocolloids like hydroxypropyl methylcellulose (HPMC), xanthan gum, and guar gum are widely used to replicate gluten's key functions in gluten‐free formulations (Akin et al. 2024; Hamdani et al. 2024; Maghsoud et al. 2024; Zhang et al. 2025).

Hydroxypropyl methylcellulose (HPMC) is a versatile polysaccharide, known for its unique property of forming thermally reversible gels upon heating, which stabilizes gas cell structures during baking and aids in moisture retention without negatively affecting the final product's texture (Crockett et al. 2011). Xanthan gum, produced by bacterial fermentation, dissolves in both hot and cold water and helps improve dough stability. Its rigid, rod‐like molecular structure contributes to high pseudoplasticity (shear‐thinning behavior) and enables the formation of a strong, elastic network crucial for gas retention and anti‐stalling effects (Encina‐Zelada et al. 2018). Guar gum, a plant‐based hydrocolloid from *Cyamopsis tetragonolobus*, is widely used as a thickening agent in the food industry. It primarily acts by increasing viscosity and water‐binding capacity and can interact synergistically with other hydrocolloids to form more entangled networks (Lee et al. 2025; Tahmouzi et al. 2023).

Although the effects of individual hydrocolloids on gluten‐free bread have been extensively studied, each hydrocolloid presents specific limitations. Increasing the amount of xanthan gum has been found to decrease the specific volume of gluten‐free bread due to increased dough elasticity and reduced extensibility, leading to a significantly firmer crumb texture (Crockett et al. 2011). In formulations using rice, maize, and quinoa flours, higher amounts of xanthan gum—despite their theoretical capacity for greater water retention—have resulted in firmer, more viscous batters with increased consistency, producing loaves with lower specific volume and a more cohesive, less springy crumb (Encina‐Zelada et al. 2018). Guar gum, while generally improving loaf volume, shows limited effect on crumb texture when used alone. However, its simultaneous incorporation with transglutaminase (TGase) has been shown to enhance crumb structure at lower enzyme concentrations, although higher doses may lead to undesirable firmness (Mohammadi et al. 2015). Additionally, combining guar gum with xanthan gum has demonstrated synergistic effects by significantly increasing dough elasticity and viscosity, thereby enhancing bread structure (Sciarini et al. 2023). Despite its benefits, guar gum has also been associated with increased water loss during storage and the development of surface cracks in fresh pasta, reducing shelf life compared to xanthan gum (Sanguinetti et al. 2015). Hydroxypropyl methylcellulose (HPMC), while functioning as a structural backbone in gluten‐free doughs, can lead to a fragile crumb when used alone, lacking resilience and springiness. These defects can be mitigated by combining HPMC with other hydrocolloids such as polyglutamic acid (PGA), which improves crumb hardness, resilience, and overall product quality (Zhao et al. 2021). The performance of hydrocolloids is also influenced by various factors, including molecular structure, concentration, flour type, interactions with other food components, and processing conditions such as temperature and pH (Zoghi et al. 2021).

Furthermore, binary hydrocolloid systems—such as xanthan gum combined with guar gum—have shown potential synergistic effects, improving batter viscosity, loaf volume, and sensory attributes like texture and flavor. Nevertheless, some combinations may negatively impact shelf life or dough rheology, such as by promoting surface cracking or altering water retention.

Therefore, this study investigates the simultaneous application of hydrocolloids—specifically HPMC, xanthan gum, and guar gum—in binary combinations to achieve synergistic effects, overcome the functional limitations of individual hydrocolloids, and improve the technological performance of gluten‐free bread. This approach offers a practical and scientifically grounded solution to persistent challenges such as low loaf volume, poor texture, and rapid staling, thereby paving the way for more acceptable and stable gluten‐free products.

## 2 Material and Methods

### 2.1 Materials

All materials, including raw quinoa (Aneed, Tehran, Iran), HPMC (Merck, Germany), sodium caseinate with 85% purity (Karoel Spice, Iran), active dry yeast (Saf‐levure, France), guar gum, xanthan gum, tapioca starch, acetylated di‐starch adipate (ADA), sodium stearoyl lactylate (SSL), corn flour, rice flour, and corn starch (purchased from Pars Khooshe Pardaz Fars, Iran), were used to produce gluten‐free bread and batter. All ingredients were selected based on their naturally gluten‐free nature and certified product labeling. No gluten‐containing grains (e.g., wheat, barley, rye, or oats) were used in the formulation. To ensure the absence of gluten, selected final bread samples were qualitatively tested using gluten detection kits, confirming gluten content was below 20 ppm, in accordance with international gluten‐free standards. Remaining common materials were obtained from the local market.

### 2.2 Methods

#### 2.2.1 Formulations

Formulations contained constant amounts of materials, and only the type of hydrocolloids varied during experiments. These base ratios were established through preliminary optimization trials to ensure suitable gluten‐free batter characteristics. Formulations contained rice flour (25%), quinoa flour (10%), corn starch (15%), tapioca starch (15%), ADA modified starch (5%), and corn flour (30%). Other formulation components, such as water (110% w/w, based on total flour and starches), margarine (5%), sugar (3%), salt (1.5%), sodium caseinate (3%), SSL (0.4%), active dry yeast (2%), and hydrocolloids (2%), were calculated as percentages of the total flour and starch weight. Hydrocolloids (2% *w*/*w* flour basis) were incorporated, a level determined from preliminary optimization trials and literature review to optimize textural and rheological properties. Their type varied, as shown in Table 1, with binary combinations equally divided (1/1 g) to facilitate the assessment of synergistic effects at a constant total hydrocolloid load. It is important to note that a control sample without hydrocolloids was not included, as preliminary trials demonstrated that such formulations lacked sufficient structural integrity to allow meaningful comparative analysis of rheological and textural characteristics.

**TABLE 1:** Gluten‐free batter and bread formulation variables. Hydrocolloids' amounts are flour/starch basis.

	Sample (100 g flour/starch basis)	HPMC (g)	Guar (g)	Xanthan (g)
1	Guar	0	2	0
2	HPMC	2	0	0
3	Xanthan	0	0	2
4	Xanthan‐Guar	0	1	1
5	HPMC‐Guar	1	1	0
6	HPMC‐Xanthan	1	0	1

*Note:* The constant base formulation, including flour, starch, and other ingredients, is detailed in the “Formulations” sub‐section.

#### 2.2.2 Bread and Batter Production

To produce the batter and bread, all flours were sieved and weighed precisely. The bread production method largely followed established protocols for gluten‐free baking (Tamilselvan et al. 2022), with some modifications adapted to our specific formulation.

The water content, determined through trial and error, was set at 110% and kept constant throughout the study. Water was used to activate the yeast and provide moisture for batter preparation. A mixer equipped with a paddle attachment was used, and the speed was set at 120 rpm during mixing steps. The batter was prepared by mixing all the ingredients for 7 min without the inclusion of hydrocolloids. Afterward, the hydrocolloids were added and mixed for an additional 4 min. After mixing, each batter batch weighed approximately 1 kg and was divided into two portions of about 300 g each prior to fermentation. The batter was then poured into pans and fermented at 35°C and 85% relative humidity until it doubled in height. Once risen, the pans were transferred to an oven and baked for 50 min at 220°C. Two replicates were carried out for all samples, which were allowed to rest for at least 3 h before further analysis.

#### 2.2.3 Rheological Properties

Batters were used for the rheological test by an MCR‐302 rheometer (Anton Paar, Austria) using a parallel plate system (25 mm diameter and 1 mm gap). Rheological analyses were performed according to standard procedures for food batters (Witczak et al. 2012). After placing the sample and covering its edges, they were given some time to relax tension and stabilize temperature. Subsequently, amplitude and frequency sweep tests were conducted at room temperature to determine the mechanical spectra and behavior of gluten‐free batter.

Amplitude sweep tests were first conducted at 1 rad/s over a stress range of 0.1–100 Pa to determine the linear viscoelastic region (LVR), where *G*′ and *G*" remained independent of applied stress. Based on this result, frequency sweep tests were then performed within the LVR at a constant strain amplitude of 0.1%, covering the angular frequency range of 1–100 rad/s. From these values, the loss tangent (tan *δ* = *G*"/*G*′), which indicates the degree of viscoelasticity, was also calculated. These frequency sweep data (*G*′ and *G*" as a function of angular frequency) were subsequently fitted to the power law model to determine parameters *K*′, *K*″, *n*′, and *n*", providing insights into the shear‐thinning behavior across the tested angular frequency range.

#### 2.2.4 Differential Scanning Calorimetry Analysis (DSC)

Differential scanning calorimetry (DSC) was performed to determine the thermal properties of the gluten‐free batters using a Mettler Toledo DSC2 STARe system (Greifensee, Switzerland). The method was adapted from previous studies (Sabanis and Tzia 2011; Witczak et al. 2012) with modifications to suit the analysis of our samples. Approximately 40 mg of each batter sample was accurately weighed into hermetically sealed aluminium pans. An empty, sealed aluminium pan was used as a reference. Samples were heated at a constant rate of 10°C/min from 25°C to 200°C.

Thermal parameters were determined using the Mettler Toledo STARe Software Version 12.00. Onset temperature (*T*_Onset_) was defined as the temperature at the intersection of the baseline and the tangent at the steepest slope of the transition curve, peak temperature (*T*_Peak_) as the temperature of maximum heat flow during the endothermic event, and end‐set temperature (*T*_Endset_) as the temperature where heat flow returned to baseline. The enthalpy change (Δ*H*) was calculated by integrating the area under the endothermic peak and expressed as Joules per gram of sample. These data were used to characterize the gelatinization behavior of the gluten‐free batters.

#### 2.2.5 Physicochemical Characteristics

**FIGURE 1:**
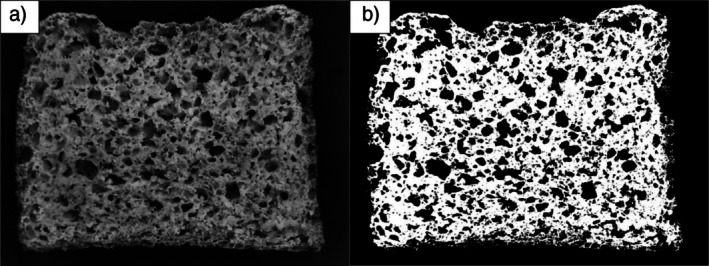
Illustration of the image analysis method used for porosity determination. (a) A representative grayscale image of the bread crumb. (b) The corresponding binary image after applying a threshold filter. The black pixels, representing the porous area, were quantified by the software to calculate the overall porosity percentage.

The physicochemical characteristics of the bread samples were determined after cooling. Ash, protein, and moisture contents were analyzed using AACC official methods 08–03.01, 46–10 (conversion factor of 6.25), and 44–01.01, respectively (AACC International 2010). Porosity was calculated by scanning the bread surface; the images were processed in 8‐bit using the Lab color system. The “Yen” thresholding algorithm was applied for differentiation (Yen et al. 1995) using ImageJ software (Schneider et al. 2012). Five measurements were taken from different parts of the breadcrumbs, and the average was reported. The image analysis process is illustrated in Figure 1.

#### 2.2.6 Color Analysis of Bread Crust and Crumb

The color of the bread's crust and crumb was analyzed. High‐resolution photographs of the samples were taken within a custom‐built, light‐controlled chamber to ensure consistent illumination. Then, the L*, a*, and b* values were determined from these photos using Adobe Photoshop software, following the method reported by Afshari‐Jouybari and Farahnaky (2011). The parameters recorded were L* (lightness, ranging from 0 for black to 100 for white), a* (representing the green‐red axis, with positive values indicating red), and b* (representing the blue‐yellow axis, with positive values indicating yellow). Six points from each sample's image were evaluated for color parameters.

It is important to acknowledge that the color determination method employed, which relies on Photoshop analysis of photographs captured in a custom‐built setup (as cited, Afshari‐Jouybari and Farahnaky 2011), may present a slight reduction in absolute accuracy compared to dedicated commercial colorimetric instruments. While this approach provides consistent relative comparisons among the samples within this study, it may narrow the scope for direct quantitative comparisons with data reported in the literature obtained via different instrumentation.

#### 2.2.7 Texture Profile Analysis (TPA)

Texture profile analysis was carried out following methods previously described by Cardinali et al. (2024) and Sahan and Ozgoren Capraz (2024), with minor modifications.

Analysis of bread samples was carried out 3, 24, and 48 h after baking by the CT‐3 texture analyzer (Brookfield Engineering Laboratories, USA). Samples were carefully prepared by cutting 15‐mm cubes exclusively from the central crumb portion of each bread slice, ensuring no crust was included in the samples. A 4500 g load was applied by a 38.1‐mm cylindrical w/rounded edge 20‐mm long probe to compress bread samples to the targeted 40% compression rate. The pretest speed was 2 mm/s while the test speed and return speed were set to 1 mm/s. Trigger load was set to be 2 g, and after this target, the attached software drew peaks. Hardness, cohesiveness, and resilience are calculated to track bread properties.

#### 2.2.8 Amino Acid Profile Analysis

Amino acid analysis was carried out similar to Barba et al. (2017). Samples were dried in an oven at 70°C until no changes in weight occurred. This step was necessary to prevent water from interfering with the hydrolysis of proteins by diluting the acids. The standard amino acid analysis method (USP < 1052 >) was used to determine amino acid profiles. High‐pressure liquid chromatography (HPLC) was performed by an Agilent 1100 (Agilent Technologies, USA) using an FLD detector. A standard amino acid‐containing kit was used to quantify amino acid contents from the graph, and results were reported in mg/kg.

#### 2.2.9 Statistics

All experiments were performed with at least three replicates. Data were analyzed and presented as mean ± standard deviation. ANOVA was conducted using IBM SPSS 26 software, followed by Duncan's post hoc test to differentiate between the means, except for the amino acid analysis, where independent t‐tests were carried out. Microsoft Excel 2017 was used to draw tables and graphs. For textural properties (hardness, cohesiveness, and resilience) presented in Figures 4, 5, 6, the data from all samples and all time points (3, 24, and 48‐h post‐baking) were analyzed together using a two‐way Analysis of Variance (ANOVA) with sample type and storage time as independent factors. This comprehensive analysis allowed us to assess the main effects of sample type and storage time, as well as their interaction. Post hoc comparisons were then performed using Duncan's test (*p* < 0.05) to identify specific significant differences among the 18 total data points (6 samples × 3 time points). The statistical groupings within the figures indicate these significant differences across all evaluated conditions.

## 3 Results and Discussion

### 3.1 Rheological Properties of Batters

The linear viscoelastic region (LVR) of gluten‐free batters was determined through amplitude sweep tests to limit deformation and prevent system breakdown. This was assessed by examining the relationship between the storage modulus (*G*'), representing the elastic characteristics, and the loss modulus (*G*"), reflecting the viscous attributes of a material. Results indicated that the batters are viscoelastic, shear‐thinning materials, which typically do not exhibit linear behavior. However, linear behavior was attained by restricting deformation to low stress levels during the early stages of testing, allowing for the investigation of both elastic and viscous properties. The LVR was selected at 0.1% strain for all samples, as this point exhibited more stable elastic moduli during testing. A constant strain of 0.1% was chosen because it fell within the linear viscoelastic region (as determined by amplitude sweep; see Section 2.2.3), ensuring that the structure of the batter was not disrupted during frequency sweep measurements.

**FIGURE 2:**
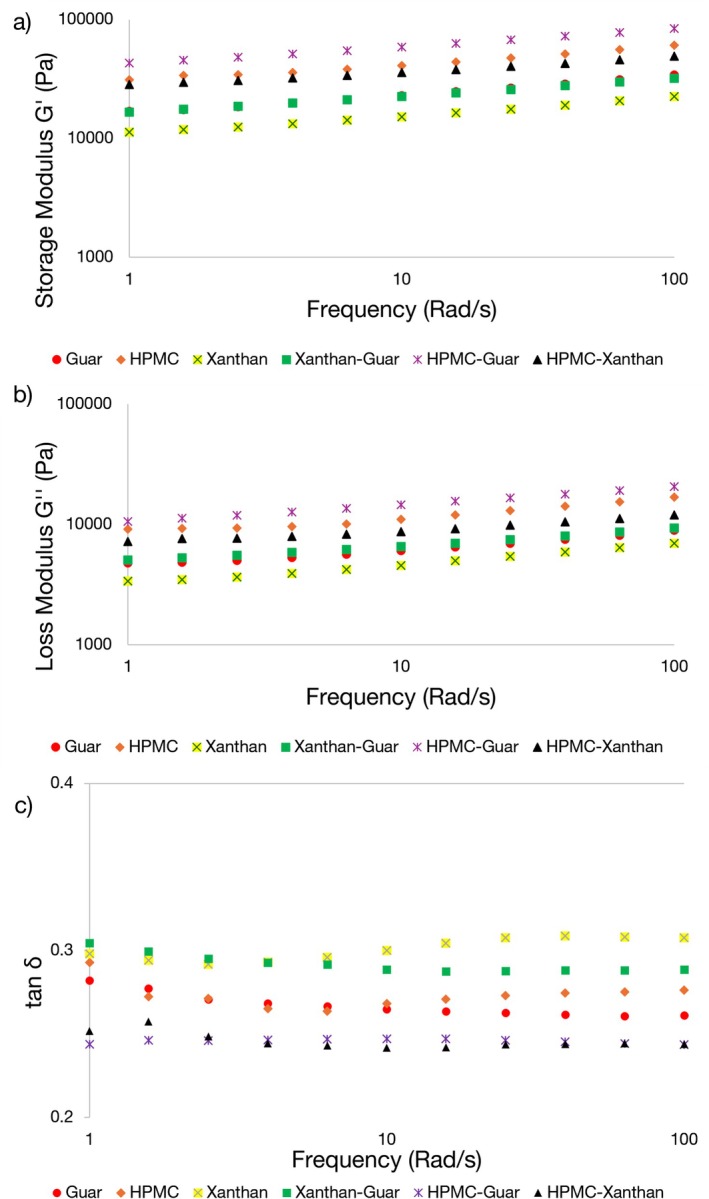
Frequency sweep results for the gluten‐free batters: (a) storage modulus (G'), (b) loss modulus (G"), and (c) loss tangent (tan *δ*) as a function of angular frequency (ω). Data for G′ and G" (a, b) are presented on a log–log scale, while data for tan *δ* (c) are presented on a semi‐log scale.

Mechanical properties were then determined within the LVR region (Figure 2). The obtained data were well‐fitted to the power law model (*R* ^2^ > 0.97). These parameters (consistency indices *K*′ and *K*″, and flow behavior indices *n*′ and *n*″), derived from frequency sweep data (*G*′ and *G*" as a function of angular frequency), quantitatively described the shear‐thinning viscoelastic behavior of the batters across the tested oscillatory range (Table 2).

**TABLE 2:** Consistency index (*K*′, *K*") and flow behavior (*n*′, *n*") values of batters obtained from the power law equation, derived from frequency sweep test data.

Sample	*K*′	*K*″	*n*′	*n*"	*R*′^2^	*R*"^2^
Guar	16.24 ± 0.08^d^	4.29 ± 0.23^e^	0.16 ± 0.00^a^	0.14 ± 0.00^c^	0.99	0.98
HPMC	29.62 ± 0.26^b^	7.83 ± 0.25^b^	0.15 ± 0.00^b^	0.15 ± 0.00^b^	0.98	0.97
Xanthan	10.88 ± 0.11^e^	3.16 ± 0.06^f^	0.15 ± 0.01^b^	0.16 ± 0.01^a^	0.99	0.99
Xanthan‐Guar	16.31 ± 0.13^d^	4.90 ± 0.04^d^	0.14 ± 0.00^c^	0.13 ± 0.00^d^	0.99	0.99
HPMC‐Guar	42.28 ± 0.31^a^	10.31 ± 0.13^a^	0.15 ± 0.00^b^	0.14 ± 0.00^c^	0.99	0.99
HPMC‐Xanthan	26.90 ± 0.15^c^	6.95 ± 0.07^c^	0.12 ± 0.00^d^	0.11 ± 0.00^e^	0.99	0.97

*Note:* Data are reported as mean ± standard deviation. Differences between values showed with different letters. The goodness of fit to the power law model (*R* ^2^) was consistently greater than 0.97 for all samples.

The results revealed that different hydrocolloid blends induced significant structural changes in the batter, with two extremes of this behavioral spectrum being clearly observed. The sample containing only xanthan gum exhibited the weakest structure, showing the lowest consistency indices (*K*′ and *K*″). This finding highlights the profound impact of the overall formulation on hydrocolloid performance. This is consistent with multiple studies that report complex and sometimes negative effects of xanthan gum. For instance, Lazaridou et al. (2007) found that while xanthan gum created an exceptionally strong dough, it resulted in a final bread with low volume and high hardness. Similarly, Schober et al. (2005) reported that increasing xanthan levels in sorghum bread decreased loaf volume and increased crumb hardness, while Hager and Arendt (2013) concluded that its performance is highly formulation dependent. In contrast, the HPMC–Guar blend created the stiffest and most rigid batter network, as indicated by the highest *K*′ value (42.28), markedly greater than all other samples (Table 2). This phenomenon can be attributed to a powerful synergistic effect, driven by intense physical entanglement between the long polymer chains of both hydrocolloids. The detrimental behavior of this blend resembles the negative impact seen for xanthan in other contexts, not only in mechanical profile but in its ability to impair final bread quality through different mechanisms (Lazaridou et al. 2007; Schober et al. 2005). It suggests a unifying principle that any hydrocolloid system that creates an excessively rigid network is likely to impair final bread quality by hindering gas expansion. This concept aligns with observations by Herawati (2019), who noted that some hydrocolloids can make the dough too rigid and stiff. This principle directly forecasts our final bread quality results, where the excessively rigid HPMC‐Guar network led to the bread with the highest measured hardness (as shown in Section 3.5).

Another notable finding was the lack of a statistically significant difference in *K*′ between the Guar and Xanthan‐Guar samples (Table 2). This absence of a synergistic or even additive effect can be attributed to competitive hydration in a water‐limited system, preventing xanthan from functioning effectively. The finding that hydrocolloid combinations do not always result in synergy was also reported by Hager and Arendt (2013) for an HPMC‐Xanthan blend.

The flow behavior indices (*n*′ and *n*") ranged from 0.11 – 0.16 for all samples (Table 2), indicating a weak gel behavior with shear‐thinning characteristics. This property is highly desirable for gluten‐free batter, as it aids in gas retention at rest while also allowing for easier processing and handling.

The predominance of elastic properties (*G*′ > *G*″) in this study suggests that the batters possessed a structured, gel‐like consistency, a crucial characteristic for retaining gas cells. The ratio *G*"/*G*' (tan *δ*) provides further insight into this balance (Figure 2). Further analysis of tan *δ* values quantifies the balance between the viscous and elastic properties and reinforces the concept of an optimal network structure (Figure 2c). All samples exhibited tan *δ* values significantly below 1.0, confirming a dominant elastic (gel‐like) character across the entire frequency range (Figure 2c).

HPMC‐Guar (0.247) and HPMC‐Xanthan (0.241) blends had among the lowest tan *δ* values compared to other formulations (Figure 2c), confirming predominantly elastic networks. The slightly lower tan *δ* of HPMC‐Xanthan, together with its considerably lower *K*′ than HPMC–Guar (Table 2), points to a more favorable balance between elasticity and flexibility, allowing greater gas cell expansion and resulting in better bread quality. In contrast, the combination of very high *K*′ (Table 2) and slightly higher tan *δ* (Figure 2c) in the HPMC–Guar blend corresponded to a more rigid and less adaptable network, leading to higher bread hardness. Therefore, tan *δ* served as a useful complementary indicator for distinguishing between elastic–flexible networks leading to high bread quality and those tending toward excessive rigidity.

Furthermore, both *G*′ and *G*" increased with angular frequency (Figure 2a,b). Comparable trends have been reported in various gluten‐free dough systems (Lazaridou et al. 2007; Sciarini et al. 2012).

While a strong, gel‐like structure (*G*′ > *G*") is essential for gas retention, the results of this section reveal a critical nuance: the degree of stiffness, as reflected primarily in *K*′ values (Table 2) and complemented by tan *δ* measurements (Figure 2c), is paramount.

As will be demonstrated in subsequent sections by correlating these rheological findings with thermal and textural properties, an excessively rigid network—such as that in the HPMC–Guar sample, which showed the highest *K*′ (Table 2) and one of the lowest tan *δ* values (Figure 2c)—can be detrimental to final product quality, whereas a more balanced network, as in the HPMC‐Xanthan blend, yields superior results.

### 3.2 Thermal Analysis of Gluten‐Free Batters

Building on the rheological findings that hydrocolloid blends created batter networks ranging from weak to excessively rigid, we next investigated the thermal properties to probe the underlying mechanisms. This analysis, using Differential Scanning Calorimetry (DSC), aimed to quantify how network strength affects the energy requirements and process of starch gelatinization. All batters exhibited a single, broad endotherm, characteristic of starch gelatinization in systems with high water availability (Li 2022). The onset temperature of gelatinization (*T*_onset_) varied significantly among the samples (Table 3). The elevated *T*_onset_ values observed in these gluten‐free systems are likely influenced by the presence of non‐starch materials, which can elevate gelatinization temperatures (Donmez et al. 2021; Xing et al. 2025). In addition, stronger hydrocolloid‐induced interactions within the batter matrix may enhance the resistance to starch gelatinization, thereby increasing the required onset temperature (Megusar et al. 2022). Similarly, the peak temperature (*T*_peak_) ranged from 108.61°C for HPMC to 115.79°C for the HPMC‐Guar blend. This elevation of gelatinization temperatures in starch–hydrocolloid systems has been widely reported (Shahzad et al. 2019; Varela et al. 2016). This increase in thermal resistance can be explained by two main mechanisms. First, hydrocolloids compete with starch for available water due to their high water‐holding capacity, which delays water migration into the starch granules. Second, the hydrocolloid network can physically entrap the starch granules, restricting their swelling. Consequently, greater thermal energy is required to overcome these barriers and disrupt starch crystallites. The end‐set temperature (*T*_endset_) ranged from 133.80°C (Guar) to a maximum of 147.84°C for the HPMC‐Xanthan blend, which was significantly higher than the values for Guar and HPMC‐Guar. This high *T*_endset_ suggests the formation of a highly stable network with reinforced structures, likely due to synergistic interactions between HPMC and xanthan, which require greater thermal energy to fully disrupt. Finally, the gelatinization enthalpy (Δ*H*), representing the total energy for the transition (Li 2022), ranged from 51.30 J/g (HPMC‐Xanthan) to 58.18 J/g (Xanthan).

**TABLE 3:** Hydrocolloids effect on the thermal properties of gluten‐free batters.

Sample	*T*_Onset_ (°C)	*T*_Peak_ (°C)	*T*_Endset_ (°C)	Δ*H* (J/g)
Guar	87.23 ± 2.01^b^	109.93 + 1.72^cd^	133.8 ± 2.86^d^	51.84 ± 1.48^c^
HPMC	85.48 ± 1.76^bc^	108.61 ± 1.85^d^	139.68 ± 2.93^c^	55.40 ± 2.32^ab^
Xanthan	93.55 ± 1.13^a^	112.47 ± 1.03^bc^	144.97 ± 2.03^ab^	58.18 ± 1.78^a^
Xanthan‐Guar	84.19 ± 1.25^c^	112.83 ± 1.50^abc^	142.95 ± 1.71^bc^	53.46 ± 1.93^bc^
HPMC‐Guar	91.88 ± 1.21^a^	115.79 ± 1.16^a^	140.67 ± 2.28^c^	52.45 ± 1.89^bc^
HPMC‐Xanthan	91.28 ± 1.07^a^	115.27 ± 2.31^ab^	147.84 ± 1.45^a^	51.30 ± 1.06^c^

*Note:* Onset temperature (*T*_Onset_), peak temperature (*T*_Peak_), endset temperature (*T*_Endset_), and the reaction enthalpy (Δ*H*) were calculated. Averages ± standard deviation reported. Different letters in same column shows significant differences (*p* < 0.05).

The observation that Xanthan sample yielded the highest enthalpy confirms its strong capacity to form a highly ordered molecular network with starch. This ability of xanthan gum to increase gelatinization enthalpy by reinforcing the starch matrix is well documented (Dahal et al. 2021; Widelska et al. 2019). It is worth noting, however, that while xanthan gum exhibited the weakest macroscopic network in rheological tests (Section 3.1), it simultaneously promoted strong molecular interactions with starch at the microscopic level, as reflected in the highest gelatinization enthalpy. This highlights the distinction between molecular‐level ordering and macroscopic structural strength.

Interestingly, the HPMC‐Xanthan blend, which exhibited the highest thermal stability (highest *T*_Endset_), recorded the lowest gelatinization enthalpy. This suggests that while the overall network created by the synergistic interaction of HPMC and xanthan is exceptionally robust, it is less crystalline in nature. The lower enthalpy may indicate a beneficial disruptive interaction, where the blend of hydrocolloids hinders the perfect re‐association of starch chains. This balance of a strong overall network with controlled starch crystallinity is often linked to improved final product qualities, such as a softer texture and reduced staling.

### 3.3 Physicochemical Properties of Bread

**FIGURE 3:**
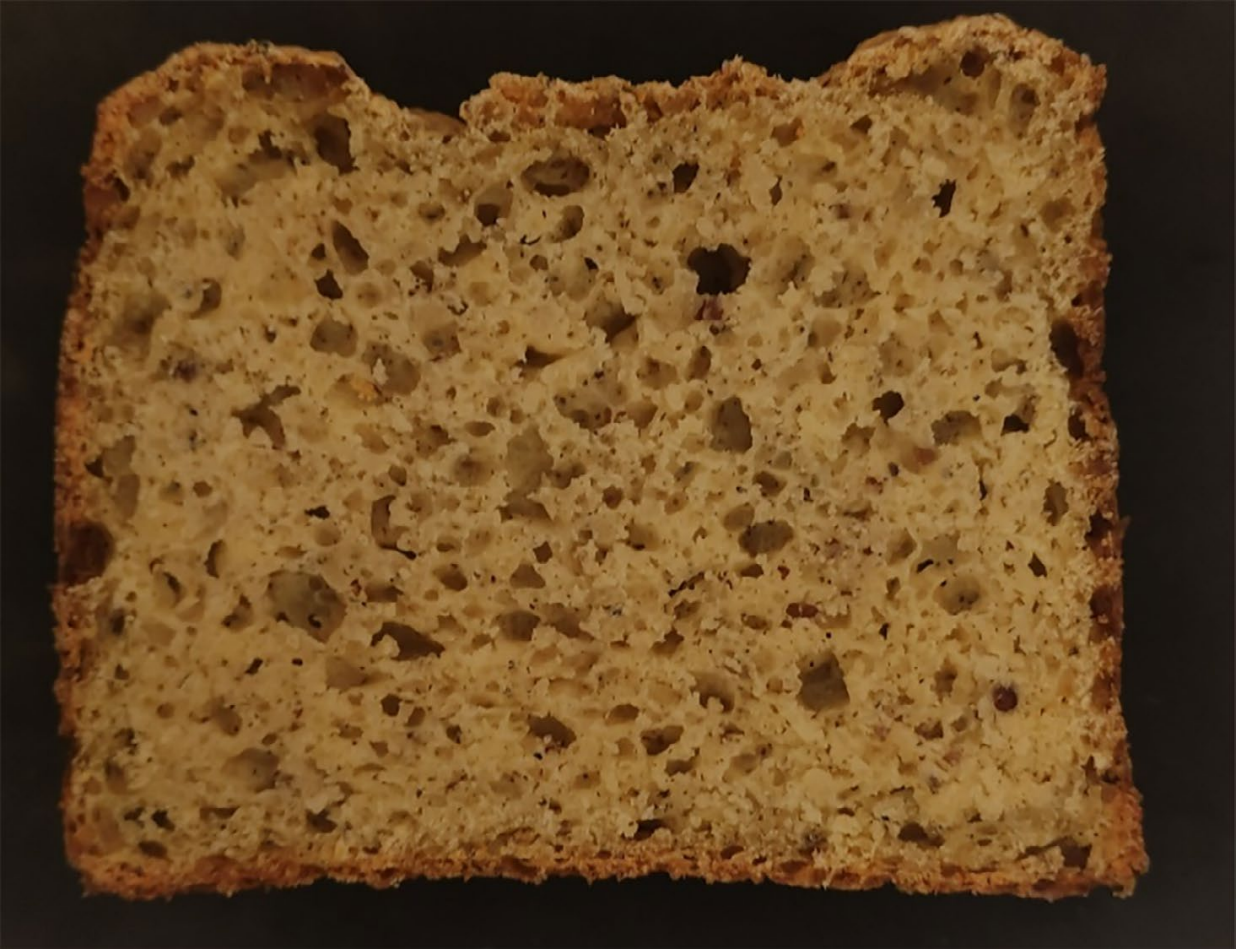
Representative gluten‐free bread formulated with HPMC–Xanthan: cross‐section showing crumb structure.

The physicochemical properties of bread (Figure 3) were evaluated, and the data are presented in Table 4. The moisture content of all samples was relatively high, ranging from 52.72% to 55.38%. This is characteristic of many gluten‐free formulations, where high water content is necessary for starch hydration and network formation (Keramari et al. 2024). The lowest moisture content was observed for the Xanthan sample, while the HPMC‐Guar blend yielded the highest. This finding directly correlates with the rheological data, where the weakest network (Xanthan) retained the least water and the strongest network (HPMC‐Guar) retained the most. While essential for initial softness, high moisture can negatively impact shelf life by promoting staling and microbial growth, highlighting a key formulation challenge (Ma et al. 2020). This phenomenon provides a direct explanation for the textural analysis (Section 3.5), in which the HPMC‐Guar sample, despite its high initial moisture, showed the most significant increase in hardness over time, confirming rapid staling.

**TABLE 4:** Moisture content, ash, protein, and porosity of gluten‐free bread samples.

Sample	Moisture content (%)	Ash (%)	Protein (%)	Porosity (%)
Guar	53.96 ± 0.17^c^	1.11 ± 0.04	5.80 ± 0.01	33.84 ± 1.89
HPMC	55.13 ± 0.23^a^	1.12 ± 0.04	5.77 ± 0.05	35.31 ± 1.95
Xanthan	52.72 ± 0.19^e^	1.11 ± 0.05	5.83 ± 0.04	34.46 ± 1.49
Xanthan‐Guar	54.53 ± 0.20^b^	1.11 ± 0.04	5.86 ± 0.07	35.45 ± 0.67
HPMC‐Guar	55.38 ± 0.20^a^	1.11 ± 0.06	5.77 ± 0.04	35.19 ± 1.30
HPMC‐Xanthan	53.39 ± 0.05^d^	1.10 ± 0.06	5.76 ± 0.05	36.04 ± 1.89

*Note:* Statistical analysis of ash content (*F* = 3.09, *p* > 0.05), porosity (*F* = 0.71, *p* = 0.63), and protein content (*F* = 2.59, *p* = 0.80) for gluten‐free bread samples, showing no significant differences across hydrocolloid treatments. Moisture content is reported as mean ± standard deviation with significance indicated by different letters.

As expected, the ash and protein contents of the breads showed no statistically significant differences among the various hydrocolloid blends. This was anticipated since hydrocolloids are polysaccharides and the protein sources in the formulation remained constant across all samples. A key finding of this study was that the porosity of the samples, a critical indicator of crumb structure and gas cell retention (Rathnayake et al. 2018), showed no significant differences among treatments, with values falling within a range commonly reported for many gluten‐free bread formulations (Evangelista et al. 2025; Sutrisno et al. 2025). This result is particularly noteworthy when considered alongside the vast differences observed in batter rheology. It suggests a complex relationship between network stiffness and final structure. For instance, the highly rigid network of the HPMC‐Guar batter (as indicated by its high *K*′) may have been too stiff to allow for optimal gas cell expansion during proofing and baking. Conversely, the much weaker network of the xanthan gum batter may have been less effective at retaining the gas produced. This could lead to different mechanistic failures resulting in a similar final porosity, suggesting that an optimal network rheology—neither too weak nor excessively rigid—is required for superior bread structure (Keramari et al. 2024). This hypothesis connects the batter properties to the final product quality and warrants further investigation. Ultimately, this structural difference is reflected in the hardness data (Section 3.5), confirming that even with similar porosity, the thicker, more robust cell walls of the HPMC‐Guar bread resulted in a significantly harder texture.

### 3.4 Crust and Crumb Color Analysis

The differences in crust color among samples were minimal for a* and b* values (Table 5), and the overall outcomes for these parameters were not statistically significant (Crust a*: *F* = 0.750, *p* = 0.598; Crust b*: *F* = 1.807, *p* = 0.162). However, crust L* values showed statistically significant differences among samples (as indicated by different letters in Table 5). This finding is particularly relevant, as gluten‐free formulations often appear pale, which may not be appealing to many consumers; thus, darker samples are generally preferred to emulate the appearance of conventional bread and enhance consumer appeal (Voučko et al. 2024).

**TABLE 5:** Effects of hydrocolloids and their blends on crust and crumb color of gluten free bread samples.

Sample	Crust			Crumb		
	L*	a*	b*	L*	a*	b*
Guar	50.50 ± 1.91^b^	22.25 ± 1.50	43.50 ± 1.29	47.25 ± 2.06^ab^	12.00 ± 0.82	41.00 ± 0.82
HPMC	44.75 ± 0.96^d^	23.50 ± 2.38	44.00 ± 1.41	46.25 ± 2.22^b^	12.75 ± 0.96	41.00 ± 1.41
Xanthan	47.50 ± 2.38^c^	24.00 ± 1.41	45.50 ± 1.00	45.75 ± 0.96^b^	12.75 ± 0.50	41.75 ± 0.96
Xanthan‐Guar	50.00 ± 3.86^bc^	23.00 ± 1.15	45.75 ± 0.96	46.25 ± 1.50^b^	12.00 ± 1.68	42.50 ± 1.29
HPMC‐Guar	53.75 ± 0.50^a^	22.00 ± 2.16	46.00 ± 1.41	49.75 ± 1.50^a^	13.50 ± 0.58	42.75 ± 0.96
HPMC‐Xanthan	48.00 ± 2.16^bc^	22.75 ± 1.50	44.25 ± 2.63	46.00 ± 2.16^b^	12.25 ± 0.96	41.25 ± 2.22

*Note:* ANOVA results for the color parameters of gluten‐free bread samples. Crust a* (*F* = 0.750, *p* = 0.598), Crust b* (*F* = 1.807, *p* = 0.162), Crumb a* (*F* = 2.147, *p* = 0.106), Crumb b* (*F* = 1.268, *p* = 0.320). No significant differences were found among the samples for these parameters (*p* > 0.05). Only significant differences found for L* and small letters showing differences among samples.

The differences in crust color among the samples can be largely explained by the varying water retention capacities of the hydrocolloid systems and their subsequent impact on browning reactions. For instance, the significant lightness of the HPMC‐Guar crust (L* = 53.75) is consistent with its superior water retention, as evidenced by the highest moisture content in the final bread (Table 4). This higher moisture likely suppressed the intensity of Maillard reactions by maintaining a lower surface temperature for a longer period during baking (Qi et al. 2025). Conversely, the darker crusts of other samples, such as Xanthan, can be linked to their lower moisture content, which facilitates faster surface drying. However, the most pronounced darkening was observed in the HPMC sample (L* = 44.75), suggesting an additional, more powerful mechanism. This is attributed to the unique thermal gelation property of HPMC. Unlike the other hydrocolloids, HPMC forms a structured gel upon heating, a process proposed to induce the migration of water from the setting matrix toward the crust's surface. This leads to particularly rapid surface drying, which in turn accelerates Maillard reactions and caramelization, resulting in more intense color development than any other formulation (Ferrero 2017).

An unexpected observation in our data was that the L* value of the crust was higher than that of the crumb for most formulations (Table 5), which is contrary to the expected outcome from Maillard reactions. This unexpected finding is likely an artifact of the image‐based colorimetry method employed. This phenomenon, known as specular reflection (Balzer and Werling 2010), can be caused by the smoother, potentially glossy surface of the crust. In this process, light from the controlled chamber reflects directly into the camera lens, creating bright spots that artificially inflate the measured L* value. In contrast, the porous and matte texture of the crumb results in diffuse reflection, leading to a more accurate color reading. Interestingly, the HPMC sample, which was the only formulation to show a logically darker crust (L* = 44.75) than crumb (L* = 46.25), may have produced a more matte surface finish. This would reduce specular reflection and yield a color value that is more representative of the actual crust color, reinforcing the hypothesis that this phenomenon is a methodological limitation rather than a chemical effect.

The crumb color of gluten‐free samples is also presented in Table 5. Only the L* value exhibited statistically significant differences between samples (as indicated by different letters), with no significant changes noted for the a* and b* parameters (Crumb a*: *F* = 2.147, *p* = 0.106; Crumb b*: *F* = 1.268, *p* = 0.320). The observed pattern can be linked to the same water management mechanisms discussed previously. For instance, the HPMC‐Guar sample, which had the highest moisture retention, also yielded the significantly lightest crumb (L* = 49.75). Conversely, formulations with lower water retention like Xanthan produced a darker crumb. The darkening effect of HPMC was also evident in the crumb, reinforcing the hypothesis that its thermal gelation mechanism influences browning throughout the entire loaf, not just on the surface.

### 3.5 Textural Properties of Bread

Evaluating textural properties such as hardness, cohesiveness, and resilience provides critical insights into the crumb structure and its evolution during storage. Gluten‐free bread is particularly susceptible to rapid staling (Šmídová and Rysová 2022), making it essential to analyze these parameters over time to understand the impact of different hydrocolloid systems.

#### 3.5.1 Hardness

**FIGURE 4:**
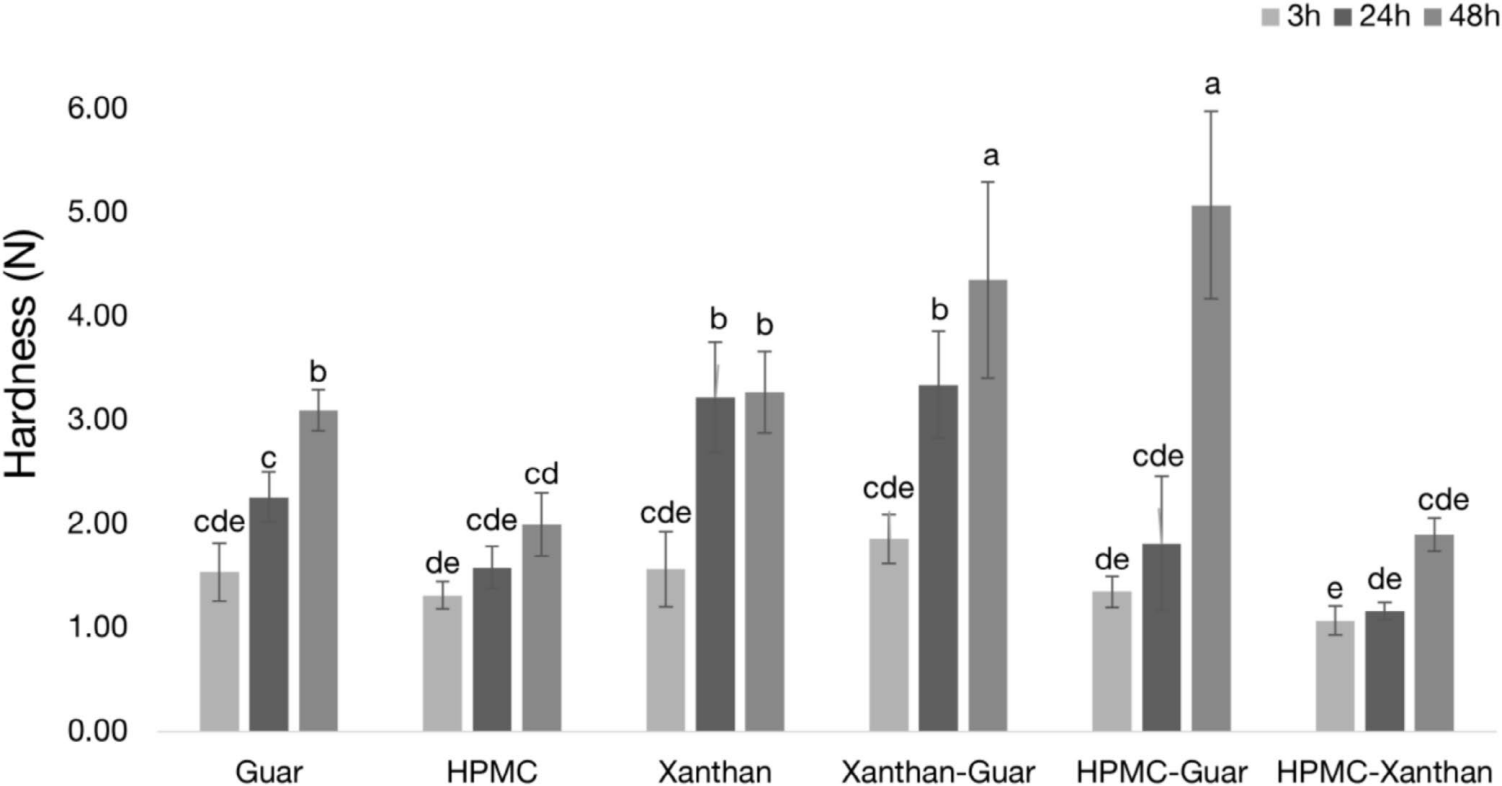
Crumb hardness of gluten‐free bread samples 3, 24, and 48 h after baking. Data are means ± standard deviation. Different letters indicate statistically significant differences (*p* < 0.05) across all samples and time points, as determined by two‐way ANOVA and Duncan's post hoc test (refer to Section 2.2.9 for detailed statistical methodology). Lower values showing softer crumb texture.

The hardness of the crumbs, a primary indicator of staling, was measured at 3, 24, and 48 h after baking (Figure 4). Hardness increased over time for all samples, confirming the progression of staling. The lowest hardness was observed in the HPMC and HPMC‐Xanthan samples, while the hardest sample was HPMC‐Guar, which reached 5.07 N after 48 h. Notably, the hardness of the HPMC‐Xanthan sample (1.90 N at 48 h) was significantly lower than that of the HPMC‐Guar sample at 48 h. The hardness of the HPMC‐Guar sample increased significantly over time, with the most substantial increase occurring between 24 and 48 h, indicating a more rapid staling rate compared to other formulations.

In contrast, the Xanthan sample showed no significant difference between the 24 and 48‐h marks, with a negligible increase from 3.22 to 3.27 N.

As anticipated from the detailed rheological and physicochemical analyses, these textural outcomes confirm a clear trend: the formulation with the most rigid batter network and highest water retention (HPMC‐Guar) produced the hardest bread, while a more balanced network (HPMC‐Xanthan) yielded a significantly softer final product.

Water content significantly affects textural properties and serves as a limiting factor in gelatinization, influencing the final texture especially when hydration levels are low (De La Hera et al. 2014). This phenomenon accelerates during storage, as bread loses moisture from the crumb to the crust, hastening the staling process (Masure et al. 2019). Therefore, it is vital to adjust both hydrocolloids and water content simultaneously, as inappropriate amounts of either can adversely affect hardness and other undesirable properties (Culetu et al. 2021).

#### 3.5.2 Cohesiveness and Resilience

**FIGURE 5:**
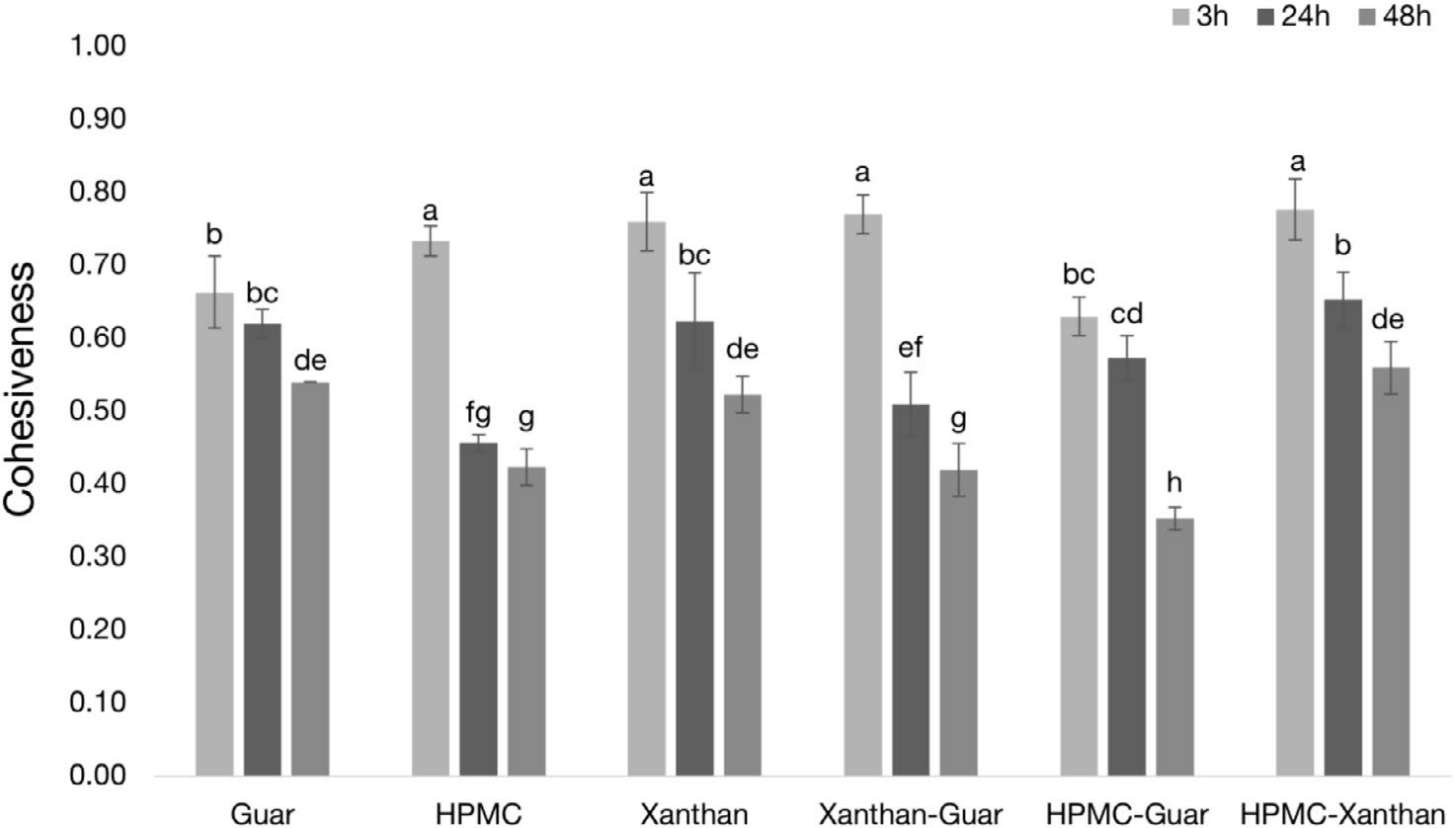
Crumb cohesiveness of gluten‐free bread samples 3, 24, and 48 h after baking. Data are means ± standard deviation. Different superscripts indicate statistically significant differences (*p* < 0.05) across all samples and time points, as determined by two‐way ANOVA and Duncan's post hoc test (refer to Section 2.2.9 for detailed statistical methodology). Higher values show better textural resistance.

**FIGURE 6:**
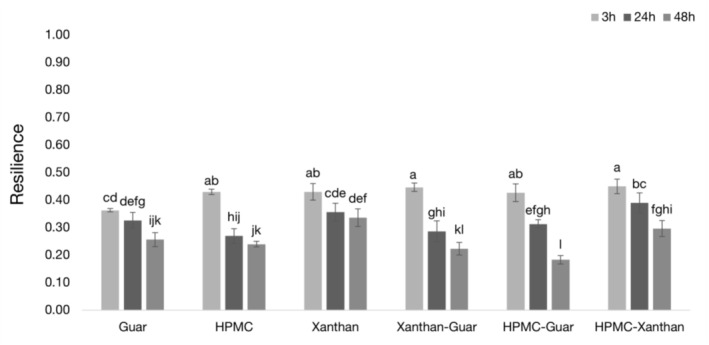
Bread samples resilience 3, 24, and 48 h after baking. Data are means ± standard deviation. Different letters indicate statistically significant differences (*p* < 0.05) across all samples and time points, as determined by two‐way ANOVA and Duncan's post hoc test (refer to Section 2.2.9 for detailed statistical methodology). Higher values are better and represent more energy absorption by the samples.

Cohesiveness and resilience are crucial indicators of crumb quality and freshness. Higher values for both are preferable as they are associated with a fresher crumb (Fadda et al. 2014; Wee et al. 2018). While high cohesiveness is generally desirable, a nuanced interpretation is necessary as it can also indicate an overly compressed structure (Koç et al. 2013). As expected, both parameters generally decreased over the 48‐h storage period for all samples, signifying a loss of structural integrity and elasticity due to staling (Figures 5 and 6).

The initial properties at 3 h were highly predictive of the final bread quality. Formulations containing xanthan gum (e.g., HPMC‐Xanthan, Xanthan‐Guar) exhibited the highest initial cohesiveness (0.78 for HPMC‐Xanthan) and resilience, indicating a well‐formed, elastic, and robust crumb structure from the outset. This aligns with literature showing that effective structure‐forming hydrocolloids are crucial for enhancing cohesiveness in gluten‐free systems (Demirkesen et al. 2010).

The HPMC‐Guar sample, however, exhibited rapid textural degradation. Despite being one of the softest samples initially at 3 h, its crumb structure was inherently flawed, showing significantly lower initial cohesiveness and resilience compared to the top‐performing groups. This combination of a soft, inelastic, and poorly bound initial crumb suggests an inefficient polymer network. This inherent weakness led to a rapid decline in quality. Over the 48‐h storage period, the HPMC‐Guar sample underwent the most dramatic transformation: its hardness drastically increased while its cohesiveness and resilience plummeted. By 48 h, it became simultaneously the hardest, least cohesive (0.35), and least resilient (0.18) sample among all conditions.

#### 3.5.3 Overall Textural Quality

Taken together, these three textural attributes—hardness, cohesiveness, and resilience—show a comprehensive and consistent picture of crumb staling. The HPMC‐Guar formulation consistently exhibited the poorest texture profile. Its rapid transformation into a hard, brittle, and inelastic crumb confirms that its overly rigid batter network, despite high water retention, is detrimental to shelf‐life. In stark contrast, the HPMC‐Xanthan sample maintained a significantly better profile, remaining softer, more cohesive, and more resilient over time. This confirms that an optimized, balanced batter network is crucial for preserving all key aspects of textural quality in gluten‐free bread.

### 3.6 Amino Acid Profile of Bread Formulation

Gluten‐free products are known for their low amino acid profiles. Given that celiac patients often experience nutritional and mineral deficiencies, it is crucial to consider the nutritional aspects of products tailored for them. A comparison of the amino acid profiles of gluten‐free bread with those of gluten‐containing bread reveals significant insights.

The amino acid profile of the technologically successful HPMC‐Xanthan formulation was compared to a commercial wheat bread (Table 6). Since hydrocolloids are carbohydrates, their substitution had a negligible effect on the amino acid content, with no significant differences observed after altering hydrocolloid blends. As expected from its higher starch content, the gluten‐free formulation showed lower levels of several essential amino acids, including leucine, isoleucine, phenylalanine, and methionine. However, the results surprisingly indicated that the gluten‐free bread contained significantly higher levels of histidine, threonine, lysine, and tryptophan than its wheat‐based counterpart.

**TABLE 6:** Amino acid content of gluten free and wheat containing bread reported in mg/kg.

Amino acid	Amino acid content (mg/kg)		Essential amino acid score	
	Wheat bread	Gluten‐free bread	Wheat bread	Gluten‐free bread
Aspartic acid	37.43 ± 1.63^b^	55.60 ± 2.43^a^	—	—
Glutamic	78.63 ± 3.19^a^	73.19 ± 4.06^a^	—	—
Serine	4.20 ± 0.30^b^	23.30 ± 1.53^a^	—	—
Histidine	1.68 ± 0.10^b^	12.47 ± 0.56^a^	0.17 ± 0.01	1.25 ± 0.06
Glycine	5.63 ± 0.31^a^	2.21 ± 0.06^b^	—	—
Threonine	4.88 ± 0.49^b^	10.5 ± 0.50^a^	0.33 ± 0.03	0.70 ± 0.03
Arginine	32.83 ± 2.24^a^	4.92 ± 0.42^b^	—	—
Alanine	36.45 ± 2.58^b^	146.2 ± 15.51^a^	—	—
Tyrosine	2.90 ± 0.26^b^	21.47 ± 0.95^a^	—	—
Methionine	9.06 ± 0.86^a^	2.98 ± 0.13^b^	0.91 ± 0.09	0.30 ± 0.01
Valine	10.63 ± 0.40^a^	9.85 ± 0.13^b^	0.41 ± 0.02	0.38 ± 0.01
Phenylalanine	11.05 ± 0.74^a^	3.52 ± 0.30^b^	—	—
Isoleucine	12.33 ± 0.96^a^	1.01 ± 0.14^b^	0.62 ± 0.05	0.05 ± 0.01
Leucine	21.00 ± 1.50^a^	5.11 ± 0.12^b^	0.54 ± 0.04	0.13 ± 0.00
Lysine	81.77 ± 8.91^b^	112.00 ± 10.82^a^	2.73 ± 0.30	3.73 ± 0.36
Tryptophan	9.70 ± 0.87^a^	11.34 ± 0.57^a^	2.43 ± 0.22	2.84 ± 0.14

*Note:* Essential amino acid score determined by dividing amino acid content to reference content: Histidine 10, threonine 15, methionine 10, valine 26, isoleucine 20, leucine 39, lysine 30, tryptophan 4.

Among these differences, the high concentration of lysine in the gluten‐free bread (112 mg/kg) compared to wheat bread (81.77 mg/kg) is particularly noteworthy for two reasons. First, from a nutritional standpoint, lysine is often the primary limiting amino acid in cereal‐based products, but it was found to be sufficient in this formulation. Second, from a biochemical perspective, lysine is one of the most reactive amino acids in the Maillard reaction (El Hosry et al. 2025). Its higher availability, therefore, likely contributes to the browning potential of the crumb and crust, providing a biochemical explanation for the color characteristics observed in Section 3.4. It is important to note, however, that the high‐water content in this gluten‐free bread keeps the internal loaf temperature below 100°C, which naturally limits the extent of this reaction (Purlis 2023).

To formally assess the nutritional quality, an amino acid score was calculated for each essential amino acid, comparing its concentration to the reference pattern for adults (World Health Organization 2007), where a score of 1.0 or higher indicates sufficiency. For the wheat bread, only tryptophan and lysine had sufficient scores (≥ 1.0), with histidine being the most limiting amino acid (0.17). The gluten‐free bread demonstrated a superior profile in this regard; not only were lysine and tryptophan sufficient, but histidine also achieved a sufficient score of 1.25. Nonetheless, scores for the remaining essential amino acids in both bread types were below one, indicating that both products have nutritional deficiencies that need to be addressed.

The sufficiency of lysine in this study is a significant finding, as other studies have identified it as a limiting factor even in fortified bread products. For instance, bread fortified with salmon powder still showed a lysine score below one (Desai et al. 2018), and high‐protein hybrid pastas failed to meet minimum requirements for several amino acids despite significant increases in lysine (Hoehnel et al. 2022). By identifying both the specific strengths (e.g., high lysine) and the remaining deficiencies of this technologically successful bread, this study provides a clear and valuable path for future research focused on targeted nutritional fortification to better meet the dietary needs of celiac patients.

## 4 Conclusion

In this study, gluten‐free batter and bread were prepared using individual hydrocolloids and their binary blends, and the effects on various properties were systematically evaluated. The results showed that the type of hydrocolloid significantly influences the properties of gluten‐free batters and breads. Textural characteristics were particularly dependent on the hydrocolloid blend. The HPMC‐Xanthan combination produced bread with lower hardness and higher resilience, while the HPMC‐Guar blend generated an overly rigid network that resulted in rapid staling. In contrast, other physicochemical and color parameters varied only slightly.

Analysis of the amino acid profile of the representative gluten‐free bread indicated that essential amino acids such as lysine were present at adequate levels, suggesting that the product provides a sound nutritional baseline in addition to acceptable technological performance. These findings highlight the importance of selecting synergistic hydrocolloid combinations, as some blends can impair quality rather than enhance it. The main limitation of this study is the absence of sensory evaluation. Therefore, to obtain a more complete understanding of product quality and address possible nutritional shortcomings, future work should validate these findings through pilot‐scale production and consumer‐based sensory trials. Moreover, additional studies should focus on incorporating protein‐rich ingredients to further improve the nutritional value of gluten‐free breads.

From an industrial perspective, the results of this work provide practical guidance. They point to hydrocolloid combinations that should be avoided, such as HPMC‐Guar, which compromise product quality, and they confirm HPMC‐Xanthan as a promising formulation for achieving better texture and extended shelf‐life. These insights reduce the risk of costly product failures and offer manufacturers a reliable basis for developing improved gluten‐free breads. Overall, this study provides a practical foundation for further innovation in commercial gluten‐free bakery products.

## Author Contributions

**Mahan Parsamajd:** data curation, writing – original draft, investigation. **Mahboubeh Fazaeli:** conceptualization, funding acquisition, supervision, writing – review and editing. **Marjan Majdinasab:** supervision, validation. **Mohammad‐Taghi Golmakani:** supervision, validation.

## Ethics Statement

This study does not involve any human or animal testing.

## Conflicts of Interest

The authors declare no conflicts of interest.

## Data Availability

The data that support the findings of this study are available from the corresponding author on reasonable request.
